# Account of Diseases in an Eastern District of London, from the 20th of May, to the 20th of June, 1801

**Published:** 1801-07

**Authors:** 


*Account of Diſeaſes in an Eaſtern Diſtrict of London, from the 20th of May, to the 20th of June,* 1801.

| | No. of Caſes. |
|---|---|
| ACUTE DISEASES. | |
| Typhus - - - - - - | 20 |
| Peripneumonia - - - - | 3 |
| Acute Rheumatiſm - - - | 5 |
| Ophthalmia - - - - - - | 2 |
| CHRONIC DISEASES. | |
| Cough - - - - - - - - | 15 |
| Dyſpnœa - - - - - - | 12 |
| Cough and Dyſpnœa - | 10 |
| Hæmoptœ - - - - - - | 3 |
| Phthiſis Pulmonalis - - | 2 |
| Anaſarca - - - - - - | 3 |
| Aſcites - - - - - - - - | 2 |
| Hydrothorax - - - - - | 2 |
| Cephalalgia - - - - - | 5 |
| Vertigo - - - - - - - - | 3 |
| Epiſtaxis - - - - - - | 1 |
| Amenorrhœa - - - - - | 4 |
| Chloroſis - - - - - - | 3 |
| Fluor Albus - - - - - | 5 |
| Diarrhœa - - - - - - - | 10 |
| Dyſenteria - - - - - - | 2 |
| Hæmorrhois - - - - - - | 4 |
| Jaundice - - - - - - | 3 |
| Hypochondriaſis - - - | 5 |
| Chronic Rheumatiſm - - | 7 |
| PUERPERAL DISEASES. | |
| Ephemera - - - - - - | 3 |
| Menorrhagia Lochialis - | 4 |
| INFANTILE DISEASES. | |
| Aphthæ - - - - - - - - | 3 |
| Herpetic Eruptions - - | 7 |
| Hooping Cough - - - | 3 |
| Vermes - - - - - - - - | 4 |
| Dentition - - - - - - | 2 |

We had the pleaſure of reporting, a few weeks ſince, that the fever, which had engaged ſo much of the attention of the medical practitioner for a conſiderable time, appeared to be on the decline, and that it was hoped it would ſoon ceaſe to make ſo conſpicuous a figure on our liſt. During the laſt two or three weeks, however, it has appeared again, and has been attended, in a number of inſtances, with ſymptoms equally violent and alarming with thoſe which have been formerly deſcribed, scribed. In some cases, after a considerable abatement of symptoms, and when the patient appeared to be in a state of convalescence, the disease has returned with an increased violence, and a fatal termination has taken place.

The very great change in the temperature of the air, for several days, during the prevalence of the north and north-westerly winds, has been productive of colds and coughs, and of an aggravated state of symptoms in some pneumonic complaints. The extreme degree of heat which was felt for a few preceding days, had produced a change of cloathing which rendered the frame more sensible to the effects produced by this change of the weather.

Disorders of the bowels, in some cases connected with fever, and in others independent of it, have been very frequent.— These may probably be attributed to the change in the temperature of the air just mentioned.

